# Research on Pneumatic Control of a Pressurized Self-Elevating Mat for an Offshore Wind Power Installation Platform

**DOI:** 10.3390/s23249910

**Published:** 2023-12-18

**Authors:** Junguo Cui, Qi Shi, Yunfei Lin, Haibin Shi, Simin Yuan, Wensheng Xiao

**Affiliations:** 1 College of Mechanical and Electrical Engineering, China University of Petroleum, Qingdao 266580, China; 2 National Engineering Research Center of Marine Geophysical Prospecting and Exploration and Development Equipment, Qingdao 266580, China; 3 Shanghai Zhenhua Heavy Industry Co., Ltd., Shanghai 200125, China

**Keywords:** wind power installation platform, pressurized self-elevating mat, pneumatic control system, fuzzy neural network PID

## Abstract

Efficient deep-water offshore wind power installation platforms with a pressurized self-elevating mat are a new type of equipment used for installing offshore wind turbines. However, the unstable internal pressure of the pressurized self-elevating mat can cause serious harm to the platform. This paper studies the pneumatic control system of the self-elevating mat to improve the precision of its pressure control. According to the pneumatic control system structure of the self-elevating mat, the pneumatic model of the self-elevating mat is established, and a conventional PID controller and fuzzy PID controller are designed and established. It can be seen via Simulink simulation that the fuzzy PID controller has a smaller adjustment time and overshoot, but its anti-interference ability is relatively weak. The membership degree and fuzzy rules of the fuzzy PID controller are optimized using a neural network algorithm, and a fuzzy neural network PID controller based on BP neural network optimization is proposed. The simulation results show that the overshoot of the optimized controller is reduced by 9.71% and the stability time is reduced by 68.9% compared with the fuzzy PID. Finally, the experiment verifies that the fuzzy neural network PID controller has a faster response speed and smaller overshoot, which improves the pressure control accuracy and robustness of the self-elevating mat and provides a scientific basis for the engineering applications of the self-elevating mat.

## 1. Introduction

The offshore wind power installation platform is a necessary piece of equipment for the installation of offshore wind turbines, and the suitability of the platform directly affects the construction efficiency of offshore wind farms, as some existing wind power installation platforms cannot adapt to deep-water operation [1,2]. Based on a project involving the construction of a 2000-ton offshore wind power installation platform, the risk characteristics of offshore platforms during the design stage have been systematically studied [3], and a significant stabilization of these platforms in terms of pitching angle and nacelle acceleration due to the installation of a gyro has been achieved [4]. Previous studies have analyzed the dynamic characteristics of offshore platforms [5,6], summarized the key technologies of offshore platforms [7], proposed that one of the most relevant cost items is the floating substructure [8], and presented the fuzzy control method as an alternative solution for the installation platform [9]. Takagi proposed an innovative jack-up seawall as an effective countermeasure to protect the port and its hinterland from tsunamis [10]. Feng designed a control strategy and algorithm according to the characteristics that the lifting platform needs to display in order to carry the test body, and realized safe and reliable remote control based on the specific motion conditions [11]. According to the working environment and system characteristics of an underwater winch, Hu et al. determined that the underwater winch was driven by a hydraulic servo, and the overall design of the hardware and software architecture of the control system was carried out [12]. Their study involved analyzing risk characteristics, stabilization with a gyro, key technologies, cost considerations, and innovative solutions for offshore wind power installation platforms.

Pneumatic control initially adopts the active pressure regulation method, and through the reasonable design of a pneumatic device, one can achieve high reliability. Wang et al. [13] first proposed the use of a programmable controller (PLC) in pneumatic control systems. Compared with relay control, PLC control has low delay and high reliability. A PLC supports multiple communication protocols and can accurately control and monitor the actuators in the process of establishing a vacuum environment [14]. Li et al. [15] designed a new multi-channel and scalable pressure controller based on STM32, which can output both positive and negative pressure. Yang et al. found that a high-performance vacuum servo system can further enhance the competitiveness of vacuum servo technology [16]. The fuzzy-PID control algorithm provides an improvement to overshoot [17], but can also adapt to the dynamic changes of the system to enhance the anti-interference performance and adaptive ability of the system, and also has a faster response speed compared with the traditional PID algorithm [18,19]. Pneumatic control has evolved from active pressure regulation to PLC-based systems, with advancements in pressure controllers and fuzzy-PID algorithms for improved precision and reliability in vacuum environments.

A robust predictive tracking control (RPTC) approach has been developed to deal with a class of nonlinear SISO systems. To improve the control performance, the RPTC architecture mainly consists of a robust fuzzy PID (RFPID)-based control module and a robust PI grey model (RPIGM)-based prediction module [20], and a self-organizing intelligent controller (SOIC) was proposed for a class of nonlinear systems [21]. An improved genetic algorithm optimization fuzzy controller has been applied to the throttle valve, which exhibits great advantages in terms of speed, stability, and robustness [22]. A simple and robust unknown dynamics estimator (UDE) was also introduced, leading to reduced computational costs and simpler parameter tuning [23]. Zhang et al. focused on two control strategies, namely fuzzy time sampled-data control and fuzzy time-space sampled-data control, and some simulations were presented to verify their effectiveness and superiority [24]. After the introduction of fuzzy rules and a neural network, the system can adjust parameters adaptively and various performances are improved [25,26]. By combining fuzzy control and artificial intelligence, the control performance of the controller can be improved [27]. Using the genetic algorithm to optimize the PID controller based on a fuzzy neural network and the genetic algorithm can improve the self-adaptive ability of the system [28]. Huang et al. proposed a new reachability analysis approach based on Bernstein polynomials which can verify that a neural-network-controlled system has a more general form of activation functions [29]. A PID-neuro-fuzzy model based on a structured artificial neural network and fuzzy rules was proposed in [30], and a multilayer structure was provided to improve the learning ability and flexibility of a fuzzy neural network in [31]. These papers introduced methods such as robust fuzzy PID and PI grey model modules, self-organizing intelligent controllers, genetic algorithm optimization, fuzzy rules, neural networks, and reachability analysis techniques to enhance control adaptability and effectiveness.

In this paper, a kind of offshore wind power installation platform with a pressurized self-elevating mat is proposed. There are relatively few studies on pneumatic control systems for self-elevating mats for wind power installation platforms. A fuzzy PID control method, optimized based on a neural network algorithm, is proposed for a self-elevating mat pressure system in this study. By combining the conventional PID algorithm with fuzzy theory, a fuzzy PID controller is designed which has the response speed of conventional PID and the self-adjusting ability of fuzzy control. The BP neural network algorithm with adaptive learning abilities is used to optimize the fuzzy PID, and the BP neural network is used to calculate the membership rule of the fuzzy control, so as to adjust the PID control parameters in real time and improve the pressure control accuracy of the self-elevating mat. This paper first innovatively designs the pressurized self-elevating mat in conjunction with the wind power installation platform, and establishes the working principle and model of the pressure control system of the pressurized self-elevating mat, and then designs the pneumatic control system and fuzzy controller. On this basis, the fuzzy neural network PID controller is designed, through the simulation of several controllers and experimental verification. To judge the control effect of each controller, we chose the best controller.

## 2. System Working Principle and Model Establishment

### 2.1. Working Principle of the Self-Elevating Mat

**Figure 1:**
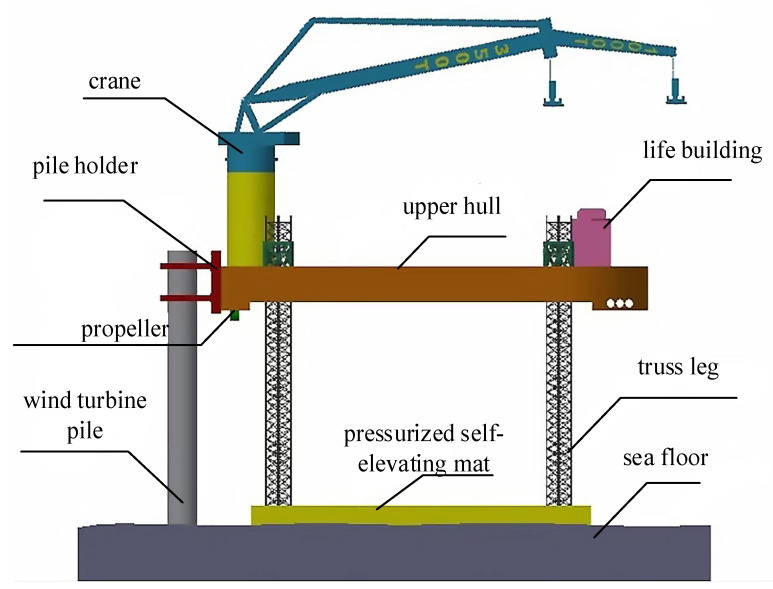
Structure diagram of wind power installation platform.

The proposed offshore wind power installation platform with a pressurized self-elevating mat is shown in Figure 1. When working, the self-elevating mat is filled with air and sits on the bottom of the seabed. However, the internal pressure system of the self-elevating mat on the platform is non-linear and delayed, and if the pneumatic control is not performed in a timely manner, it will cause potential safety hazards for the installation platform. Therefore, research on the pneumatic control technology of self-elevating mats is of great significance to avoiding damage to the self-elevating mat structure and improving the safety of the platform. The concept of a pressurized self-elevating mat is proposed for the first time in this study. Because the self-elevating mat for a wind power installation platform is large, complex, has many actions, and is difficult to control, it is a key innovation point to design the inlet pressure control system of pressurized self-elevating mats by using a fuzzy neural network PID controller.

The self-elevating mat is a closed area, and its surface is connected with gas pipelines and other components. There is also a set of pinion and rack mechanism system installed on the truss legs. During the operation of the wind power installation platform, the pressurized self-elevating mat is filled with seawater and sent to the seabed via the pinion and rack mechanism. Then, high-pressure gas is pumped into the mat for drainage. At this time, the mat filled with air obtains buoyancy, which is transmitted to the upper hull through the truss leg to balance the platform’s own weight, and carries out the operation of lifting the upper hull combined with the seabed reaction.

The offshore wind power installation platform is mainly composed of the upper hull, the pressurized self-elevating mat, and four truss legs. The upper hull is equipped with a crane, pile holder, propeller, and other facilities.

**Figure 2:**
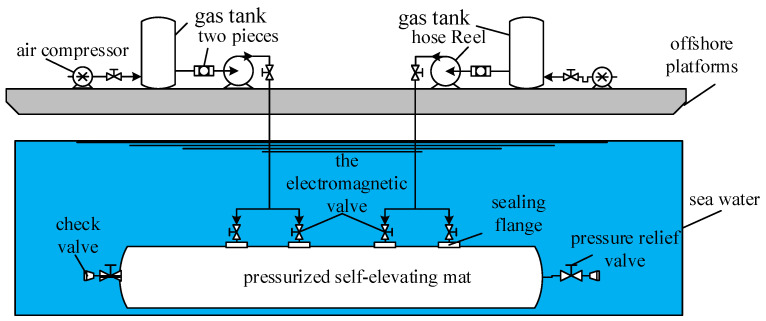
Air supply flow chart of the air source device.

The pneumatic control system of the pressurized self-elevating mat is shown in Figure 2, and is mainly composed of the air compressor, gas storage tank, and pipe winding machine on the upper platform, and the solenoid valve, pressure relief valve, and check valve on the self-elevating mat. When the wind power installation platform is in operation, the pressurized self-elevating mat filled with water is required to sit on the seabed. High-pressure gas is generated by the external pressurized equipment and then sent to the self-elevating mat through the gas transmission pipeline. The seawater in the self-elevating mat is discharged and buoyancy is generated, meaning that the upper platform is lifted to realize wind power installation.

There are three main stages in the work process of the wind power installation platform:

Sinking and drainage process. When the wind power installation platform reaches the work site, the self-elevating mat is sunk through the platform pile leg rack lifting mechanism. In this process, the valves around the self-elevating mat are opened to connect the seawater inside and outside the self-elevating mat box, and there is no pressure difference. When the self-elevating mat is completely seated on the seabed, the pneumatic device is used to fill the inside of the self-elevating mat with high-pressure gas and the seawater in the box is discharged. The buoyancy is generated to lift the upper platform through the spud legs.

Washing out pile and lifting process. The self-elevating mat needs to be returned to the upper platform after the operation finishes. Due to the large adsorption force between the self-elevating mat and the seabed ballast, the pile punching device is required to drive the medium between the self-elevating mat and the seabed surface in order to reduce the adsorption force. At the end of the punching pile, the self-elevating mat is lifted into the upper hull through the pile leg. In order to reduce the force acting on the pile leg, the self-elevating mat is lifted up in a hollow state.

Pressure retaining process. During the self-elevating mat’s seating and rising, in order to prevent the structure being crushed by seawater, it is necessary to ensure that the box is under positive pressure (the internal pressure is greater than the external pressure), and the internal and external pressure difference is about one atmosphere. At the same time, it is necessary to monitor the internal pressure and compensate the pressure in order to avoid changes in the internal pressure of the self-elevating mat due to sealing problems or a local leakage.

### 2.2. Modeling of the Pressure System

(1) Intake model

The throttling element of the pneumatic control system utilizes an electromagnetic proportional valve with a fast-opening characteristic. The relationship between the opening degree and the flow coefficient can be expressed as:

(1) $$K_{v}=-1.0741e^{-\frac{x}{3874.6}}+1.0816$$

This function is fitted by origin using the polynomial method, where *x* is the opening size, 0~100%, and *K_v_* is the flow coefficient when the opening is *x*, 0~100%.

The mass flow rate of the solenoid valve is determined by the valve flow coefficient and the inlet–outlet pressure difference, so the mass flow rate of the solenoid proportional valve into the self-elevating mat is [32]:

(2) $$M_{m}=C_{p}K_{v}xP_{1}\sqrt{\frac{2}{RT}}{\left(\frac{2}{K+1}\right)}^{\frac{1}{K-1}}\sqrt{\frac{K}{K+1}}$$

where *M_m_* is the mass flow rate of the valve, kg/s; *C_p_* is the leakage coefficient, which is 0.6~1.0; *P*_1_ is the inlet pressure of the solenoid valve, Pa; *T* is the temperature, K; *K* is the adiabatic index, which is 1.4; and *R* is the gas constant, 8.314 J/(mol·K).

(2) Exhaust model

The self-elevating mat in this study is a closed area, and its surface is connected with the gas transmission pipeline and other components. In actual engineering, the greater the airtightness of the container is, the higher the construction difficulty and cost are. Therefore, a small amount of leakage is allowed in the construction process of the self-elevating mat, but the leakage process must meet the requirements of establishing a pressure-maintaining control system for the self-elevating mat. That is, gas discharge of the self-elevating mat mainly consists of two parts: leakage and pressure relief valve exhaust. Because the gas velocity during leakage is subsonic, the formula for calculating the leakage mass *M_T1_* is as follows [33]:

(3) $$M_{T1}=C_{p}A\rho_{i}\sqrt{\frac{2K}{K-1}RT\left[{\left(\frac{P_{2}}{P_{i}}\right)}^{\frac{2}{K}}-{\left(\frac{P_{2}}{P_{i}}\right)}^{\frac{K+1}{K}}\right]}$$

where *A* is the leakage area, m^2^; *ρ_i_* is the air density at time *t* of the box, kg/m^3^; and *P_i_* is the pneumatic pressure at box *t*, Pa.

The pressure relief flow characteristics of the pressure relief valve are largely similar to the leakage process, but the pressure relief area is controlled by the current, so the mass flow rate *M_T2_* flowing through the pressure relief valve is calculated as follows:

(4) $$M_{T2}=C_{p}K_{v}xA\rho_{i}\sqrt{\frac{2K}{K-1}RT\left[{\left(\frac{P_{2}}{P_{i}}\right)}^{\frac{2}{K}}-{\left(\frac{P_{2}}{P_{i}}\right)}^{\frac{K+1}{K}}\right]}$$

(3) Transfer function establishment

The intake and exhaust flow of the self-elevating mat are obtained, and so the complete flow equation of the gas mass *M_c_* in the box is as follows:

(5) $$M_{c}=M_{m}-M_{T1}-M_{T2}$$

According to the continuity equation, it can be seen that the inflow and outflow of gas inside the self-elevating mat are equal to the change rate of gas mass, and the power source for discharging gas mainly comes from the internal and external pressure difference. This means that the discharged mass can be linearly converted, and Equation (5) can be expressed as:

(6) $$\frac{V}{KRT}\cdot \frac{dP}{dt}=k_{m}x-k_{1}p-k_{2}p$$

(7) $$k_{m}=C_{p}K_{v}x\sqrt{\frac{2}{RT}}{\left(\frac{2}{K+1}\right)}^{\frac{1}{K-1}}\sqrt{\frac{K}{K+1}}$$

where the *k_m_* in (6) is calculated in (7)**,** where *k_1_* is the flow pressure coefficient at the leak and *k_2_* is the flow pressure coefficient of the pressure relief valve.

The pneumatic control system of the self-elevating mat controls the pressure change inside the self-elevating mat by controlling the current. The actuator is a solenoid valve, and the controlled object is the internal pressure of the self-elevating mat. The input of the solenoid valve is the control current, and the output is the valve opening. This process can be regarded as a second-order link, and the transfer function can be expressed as [34]:

(8) $$\frac{x(s)}{i(s)}=\frac{\frac{k_{v}}{K_{v}}\omega_{v}^{2}}{s^{2}+2\xi_{v}\omega_{v}s+\omega_{v}^{2}}$$

where *k_v_* is the gain coefficient of the solenoid valve, m·A^−1^; *ω_v_* is the natural frequency of the solenoid valve, rad·s^−1^; and *ξ_v_* is the damping ratio of the solenoid valve.

Under zero initial conditions, the Laplace transform of Equation (6) is carried out, and the transfer function between the pressure inside the self-elevating mat and the valve opening is as follows:

(9) $$\frac{P(s)}{x(s)}=\frac{k_{m}}{\frac{V}{KRT}\cdot s+k_{1}+k_{2}}$$

The solenoid valve opening x is taken as the input in the self-elevating mat link, and the internal pressure of the self-elevating mat is taken as the output. Considering the delay of the solenoid valve, the transfer function of the self-elevating mat pneumatic control system can be obtained by incorporating the relevant parameters:

(10) $$G(s)=\frac{P(s)}{x(s)}\cdot \frac{x(s)}{i(s)}=\frac{k_{m}k_{v}\omega_{v}^{2}}{K_{v}\left(\frac{V}{KRT}\cdot s+k_{1}+k_{2}\right)\left(s^{2}+2\xi_{v}\omega_{v}s+\omega_{v}^{2}\right)}e^{-\tau s}$$

## 3. Design of a Fuzzy PID Controller for a Pneumatic Control System

When regulating the internal pressure of the self-elevating mat, the PLC first collects the value of internal pressure and the status of each valve, calculates the deviation between the set pressure and the actual pressure, controls the opening of each valve using the controller to control the gas flow, and realizes the adjustment of the internal pressure of the self-elevating mat. The self-elevating mat of the wind power installation platform has a large volume, a long transmission pipe, and high pressure requirements. Additionally, the self-elevating mat has a large pressure change range and a long pressure control time under different operating conditions. Therefore, it is necessary to meet the gas demand of the self-elevating mat under different operating conditions and ensure that the pressure in the chamber stays positive and quickly reaches the set value, which becomes a difficult problem in the design of pneumatic control systems. In this paper, the fuzzy control theory is used to design the controller, and the neural network is used to optimize the fuzzy controller.

### 3.1. Fuzzy Control Algorithm

Fuzzy control, using expert experience to make fuzzy rules, can quickly adjust the system control parameters in order to ensure that the system maintains the best control level. By combining the conventional PID algorithm with fuzzy theory, a fuzzy self-tuning PID controller is designed which not only has the high precision and fast response speed of conventional PID control, but also has good robustness and a parameter-adaptive function, so as to achieve the goal of high-quality control and energy savings.

**Figure 3:**
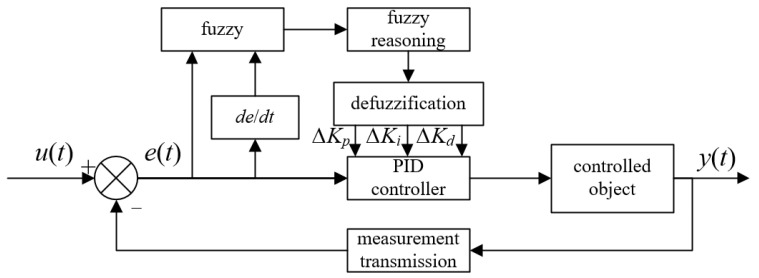
Schematic diagram of fuzzy PID control.

Fuzzy PID can achieve a better control effect according to fuzzy rules, aiming to address the nonlinear and complex model problem of solenoid valve opening flow in the pneumatic control system for a pressurized self-elevating mat. The core of fuzzy PID is the design of the fuzzy controller [35]. The principle of fuzzy PID control is shown in Figure 3. By fuzzifying the input and applying fuzzy reasoning and defuzzification, the correction value of PID parameters is determined, and the parameters of the PID controller are adjusted in real time to improve the control accuracy.

(1) Fuzzifying

In order to obtain a better membership function, the selection should be combined with the characteristics of the system. In this paper, the input adopts a linear trigonometric function to describe the membership function, while the relationship between the current and the opening of the controlled valve is non-linear, and so the output is described by a Gaussian function.

**Figure 4:**
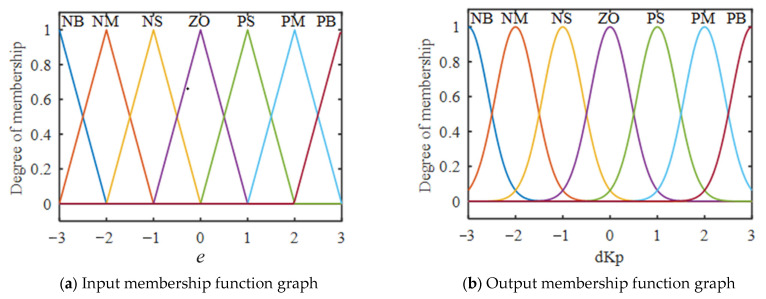
The membership function of *e* and *ec*.

In Figure 3, the input of the fuzzy controller is the pressure deviation *e*, the rate of change *ec*, and the output is the correction values of PID parameters Δ*K_p_*, Δ*K_i_*, and Δ*K_d_*. In this paper, the fuzzy domain is divided into subsets (NB, NM, NS, ZO, PS, PM, and PB), and the fuzzy subset corresponds to the uniformly quantized domain [−3, 3]. Combined with the characteristics of the pneumatic control system, the input is a kind of membership function described by a linear trigonometric function. Meanwhile, the current of the controlled valve has a nonlinear relationship with the opening, and so the output is described by a Gaussian function. The membership function images are shown in Figure 4.

(2) Fuzzy reasoning

The fuzzy reasoning method adopts the Mamdani model to establish the fuzzy PID, and combines expert experience to formulate the relationship between input state quantity [*e*, *ec*] and control variable [Δ*K_p_*, Δ*K_i_*, Δ*K_d_*]. A total of 49 inference rules are shown in Table 1, Table 2 and Table 3.

(3) Defuzzification

Defuzzification is the process of converting the abstract value result obtained through fuzzy reasoning into a clear value that can be recognized by the actuator through various methods [36]. There are many ways to de-blur, and the calculation method should be reasonably selected according to the control requirements of the pneumatic control system. Commonly used methods include the maximum degree of membership method, the weighted average method, and the center of gravity method. For the center of gravity method, the system output is the barycenter of the area surrounded by the membership function curve and the coordinate axis. The center of gravity method can respond to the input signal even when the input signal does not change much, and the value is smooth and has high precision. Therefore, the center of gravity method is used for defuzzification. The calculation formulas are shown in (11).

(11) $$z_{0}=\frac{\int_{a}^{b}z\mu_{c}(z)dz}{\int_{a}^{b}\mu_{c}(z)dz}$$

After the clear Δ*K_p_*, Δ*K_i_*, and Δ*K_d_* are obtained, they should also be adjusted through combination with the actual PID parameters, as shown in Equation (12):

(12) $$K_{x}=K_{x0}+k_{x}\cdot \Delta K_{x}$$

where *K*_*x*0_ is the initial value of each PID parameter; *k_x_* is the quantization factor; and Δ*K_x_* is the correction value after sharpening.

### 3.2. Simulation of Control Algorithm

**Figure 5:**
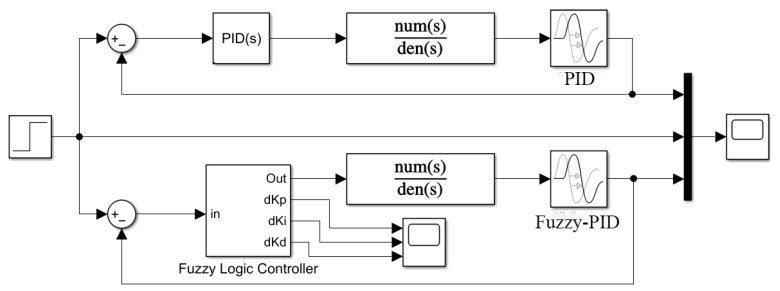
Simulation structure of the fuzzy PID controller.

**Figure 6:**
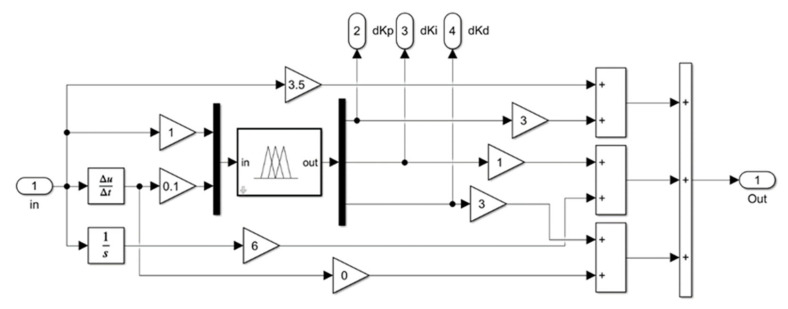
Internal structure of the fuzzy controller.

In the previous section, the self-adjusting fuzzy PID controller was analyzed and designed for the pneumatic control system. In this section, the controller is built using Simulink and simulation experiments are carried out. The fuzzy PID simulation model is shown in Figure 5, and the internal structure of the fuzzy controller is shown in Figure 6.

**Figure 7:**
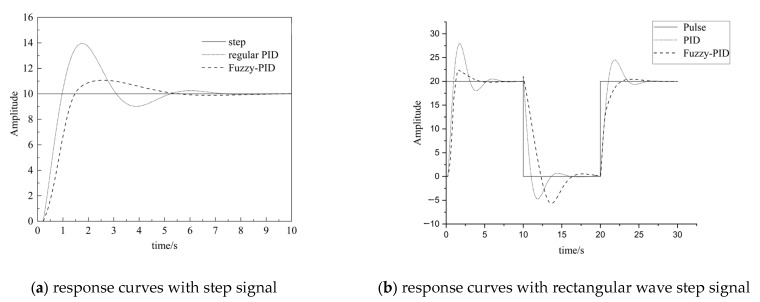
Fuzzy PID simulation.

The inputs of the system are set as the step signal and rectangular wave step signal, respectively, with an amplitude of 10, the simulation times are 10 s and 30 s, respectively, and the three initial parameters of PID are *K_p_* = 3.5, *K_i_* = 6 and *K_d_* = 0, respectively. With the same PID parameter setting, conventional PID control and fuzzy PID control were used to simulate and analyze the pneumatic control system according to the structure in Figure 5, and the response curves are shown in Figure 7a,b.

It can be seen that, when the input is set as step signal, the rise time of the traditional PID is 0.875 s, the peak value is 13.96, and the stability time is 5.085 s. Meanwhile, the step response rise time of the fuzzy controller is 1.271 s, the peak value is 11.07, and the stability time is 4.865 s. The comparison shows that the fuzzy control has a smaller overdrive and a faster stabilization time. When the input is set as rectangular wave step signal, it can be seen that the fuzzy control has a smaller overdrive but the stabilization times are very close to each other.

**Figure 8:**
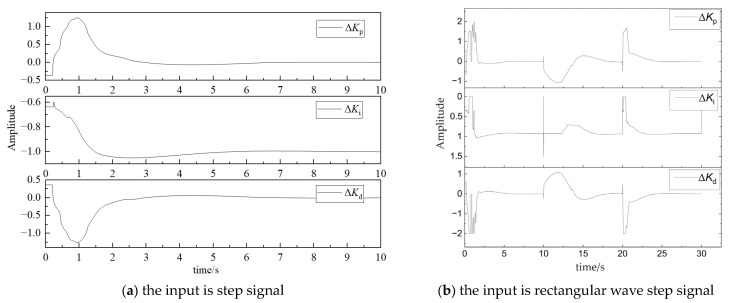
Fuzzy control parameter results.

The output parameters Δ*K_p_*, Δ*K_i_*, and Δ*K_d_* of the fuzzy controller are shown in Figure 8a and b, respectively.

**Figure 9:**
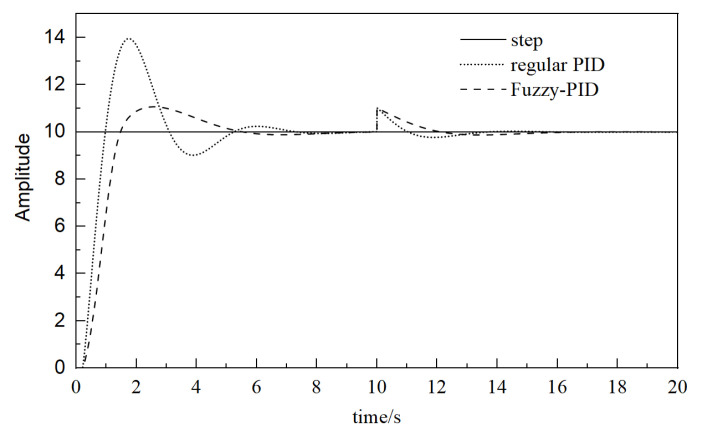
Simulation analysis of interference introduction.

In the pneumatic control system, the feedback of the system or other signals will change due to various accidents, which will cause interference. It is necessary to analyze the anti-interference ability of the system. During the simulation, at 10 s, an interference with an amplitude of 1 is introduced, and the overall simulation time is set as 20 s. The simulation results are shown in Figure 9.

It can be seen from the simulation results that although the response of fuzzy control is more stable than that of conventional PID, the response speed is slower when dealing with interference. In addition, the response speed of fuzzy PID is slow in the initial stage, and the adjustment ability in case of interference is poor, so it is necessary to introduce an artificial intelligence control method to improve fuzzy control.

## 4. Design of a Fuzzy Neural Network PID Controller for a Pneumatic System

### 4.1. Fuzzy Neural Network Algorithm

The fuzzy rules and membership function of the fuzzy controller are generally obtained according to expert experience. However, for a controlled object with interference, better fuzzy rules and membership functions can no longer be obtained by means of expert experience, and there are problems such as self-learning. Therefore, the BP neural network is introduced to optimize the fuzzy controller. By combining a neural network and fuzzy PID control, a PID controller based on a fuzzy neural network is formed, and PID parameters are adjusted using the adaptive fuzzy neural network to increase system stability.

**Figure 10:**
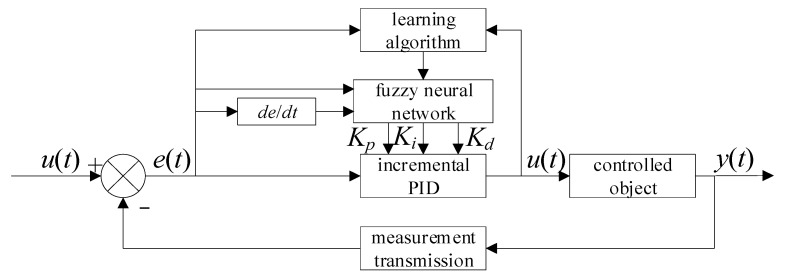
Schematic diagram of FNN-PID control.

The input of the fuzzy neural network PID (FNN-PID) controller is the pressure error *e* and rate of change *ec*, and the membership rules are adjusted in real time through the learning algorithm, while the three parameters of PID are the output. The control principle is shown in Figure 10.

(1) FNN-PID controller design

**Figure 11:**
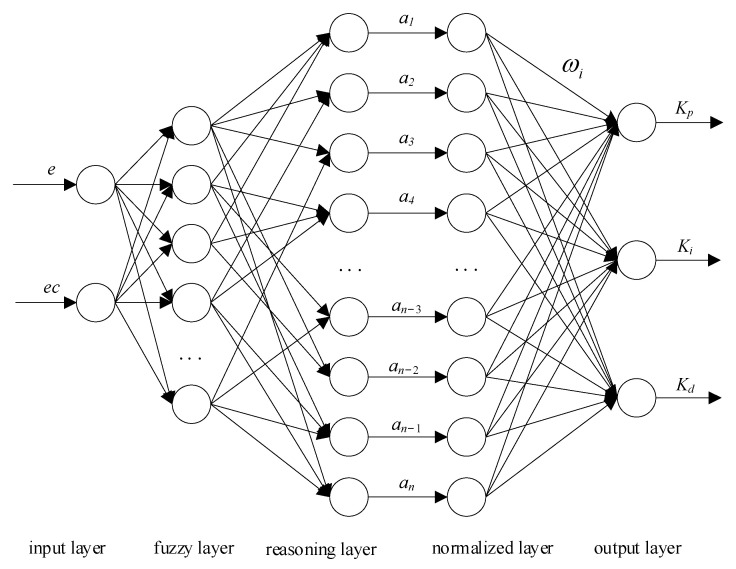
Fuzzy neural network structure.

In this paper, the FNN-PID controller is designed based on the Mamdani model, which is a five-layer feedforward approximation network with two inputs and three outputs. The inputs in the input layer are the deviation *e* and the rate of change *ec*. After the membership degree fuzzification layer and fuzzy inference layer are normalized in the fuzzy neural model, the output layer performs clear calculations based on the previous layer to obtain the corresponding PID control parameters *K_p_, K_i_*, and *K_d_*. The fuzzy neural network structure is shown in Figure 11.

The first layer is the input layer, whose input vector *x_i_* = [*e*, *ec*]^T^, and this layer has two nodes. The input and output of this layer can be expressed as:

(13) $$\left\{{}_{O_{ij}^{\left(1\right)}=I_{i}^{\left(1\right)}}^{I_{i}^{\left(1\right)}=x_{i}}\right.$$

where *I* is the input of the network layer; *O* indicates network layer input; (1) is the first layer of the network; *x_i_* is the input of the ith node, *I* = 1, 2; and *j* is the output node of the layer network, *j* = 1, 2, ..., 7.

The second layer is the fuzzifying layer. The input is fuzzified and the membership function is determined using a Gaussian function. The fuzziness domain of each input vector is divided into NB, NM, NS, ZO, PS, PM, and PB, which are −3, −2, −1, 0, 1, 2, and 3, respectively. Each node represents one of the fuzzy subsets, and there are 14 nodes in total. The input and output of this layer can be expressed as:

(14) $$\left\{\begin{array}{l} I_{i}^{\left(2\right)}=O_{ij}^{\left(3\right)}\\ O_{ij}^{\left(2\right)}=u_{ij}(x_{i})=\exp\left[-\frac{{\left(x_{i}-c_{ij}\right)}^{2}}{b_{ij}^{2}}\right] \end{array}\right.$$

where *c_ij_* is the central value of the membership degree function and *b_ij_* is the width of the membership function.

The third layer is the fuzzy reasoning layer. All nodes in the hidden layer have corresponding fuzzy rules. Based on the matching of fuzzy rules, the fitness of each $u_{ij}(x_{i})$ value is calculated. The input and output of this layer can be expressed as:

(15) $$\left\{\begin{array}{l} I_{i}^{\left(3\right)}=O_{ij}^{\left(2\right)}\\ O_{l}^{\left(3\right)}=\prod_{i=1}^{2}\prod_{j=1}^{7}u_{ij}(x_{i}) \end{array}\right.$$

where *l* = 1, 2, ..., 49.

The fourth layer is the normalization layer, which limits the results to a certain range and speeds up the convergence rate of the fuzzy neural net. The input and output of this layer can be expressed as:

(16) $$\left\{\begin{array}{l} I_{l}^{\left(4\right)}=O_{l}^{\left(3\right)}\\ O_{l}^{\left(4\right)}=\frac{I_{l}^{\left(4\right)}}{\sum_{l=1}^{49}I_{l}^{\left(4\right)}} \end{array}\right.$$

The fifth layer is the output layer, which has three nodes. Through the clear calculation of the input of the fourth layer, the final output of the controller and the three control parameters of PID are obtained. The input and output of this layer can be expressed as:

(17) $$\left\{\begin{array}{l} I_{l}^{\left(5\right)}=O_{l}^{\left(4\right)}\\ O_{h}^{\left(5\right)}=\sum_{h=1}^{3}\sum_{l=1}^{49}\omega_{hl}I_{l}^{\left(5\right)} \end{array}\right.$$

where *h* is the output number, which is 1,2, or 3, and *ω_hl_* is the connection weight between the fourth and fifth layers.

(2) BP learning algorithm

After establishing the fuzzy neural network structure model, it is necessary to modify the central value of the membership function *c_ij_*, width *b_ij_*, and the connection weight *ω_hl_* between the fourth and fifth layers. The performance index function *E*(*k*) of the learning algorithm is:

(18) $$E(k)=\frac{1}{2}e{\left(k\right)}^{2}=\frac{1}{2}{\left[u(k)-y(k)\right]}^{2}$$

where *u*(*k*) is the set value of the pressure control system at the KTH iteration; *y*(*k*) is the output value; and *e*(*k*) is the error.

In order to reduce the control error of the system, the minimum value of fuzzy neural network parameters is searched for through the algorithm gradient. The iterative formulas of each network parameter are as follows:

(19) $$\omega_{ij}(k+1)=\omega_{ij}(k)-\eta \frac{\partial E}{\partial \omega_{ij}}+\alpha \left(\omega_{ij}(k-1)-\omega_{ij}(k-2)\right)$$

(20) $$c_{ij}(k+1)=c_{ij}(k)-\eta \frac{\partial E}{\partial c_{ij}}+\alpha \left(c_{ij}(k-1)-c_{ij}(k-2)\right)$$

(21) $$b_{ij}(k+1)=b_{ij}(k)-\eta \frac{\partial E}{\partial b_{ij}}+\alpha \left(b_{ij}(k-1)-b_{ij}(k-2)\right)$$

where *η* is the learning rate and *α* is the momentum factor.

(3) Establishment of FNN-PID controller

After the mathematical model and the learning algorithm of the fuzzy neural network are determined, the incremental PID is introduced into the fuzzy neural network PID controller. Incremental PID only outputs the latest three iterations results. If the system sampling problem occurs, the output of the system will not be affected in the short term. The stability of the system can be increased.

The rule of incremental PID control is shown in Equations (22) and (23):

(22) $$\Delta u(k)=K_{p}\left[e(k)-e(k-1)\right]+K_{i}e(k)+K_{d}\left[e(k)-2e(k-1)+e(k-2)\right]$$

(23) u(k)=u(k−1)+Δu(k)

where *u*(*k*) and *e*(*k*) are the controller output and control error of the system during *k* iterations, respectively, and *K_p_*, *K_i_*, and *K_d_* are the outputs of the fuzzy neural network model.

### 4.2. Simulation and Result Analysis

**Figure 12:**
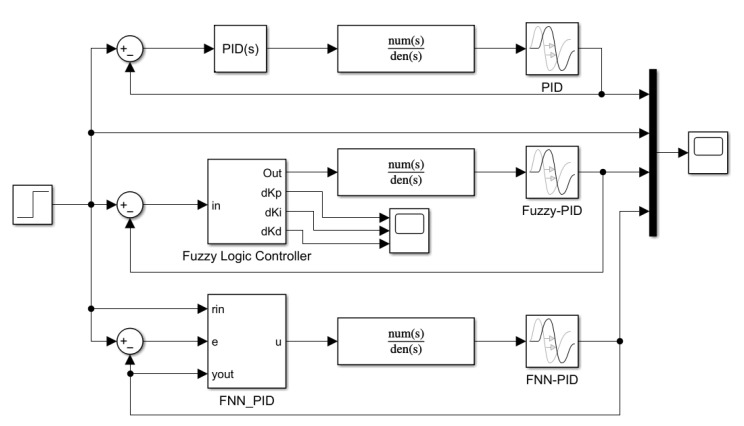
FNN-PID controller simulation structure.

According to the design of the fuzzy network controller in the previous section, a fuzzy neural network PID controller model is established. The S-function is adopted to realize the function of the fuzzy neural network controller. The input of the controller includes set values, deviation, and the output pressure value of the pneumatic system, in which the output is the PID output optimized via the fuzzy neural network. The simulation model is shown in Figure 12.

**Figure 13:**
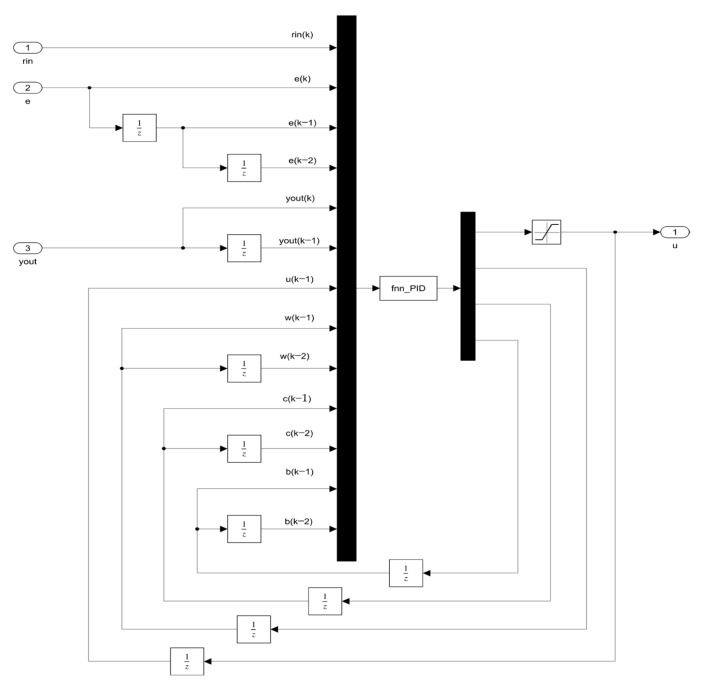
FNN-PID controller.

The internal structure of the FNN-PID controller is shown in Figure 13. It can be seen that the controller inputs the deviation obtained by the delayed sampling module, the relevant parameters of the membership function and other values at different sampling times into the fuzzy neural network PID control system established by the S-function. Then, the function of the fuzzy neural network PID is carried out.

**Figure 14:**
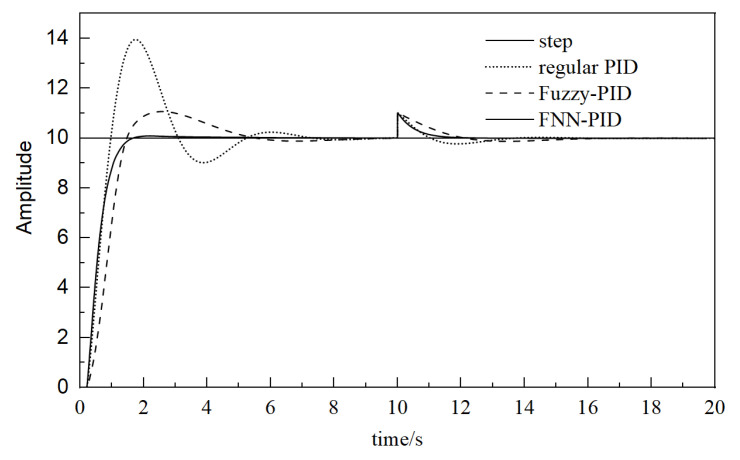
Simulation results under interference.

Using the same simulation time and input value, and introducing interference sources, the simulations of the pneumatic system controlled by conventional PID, fuzzy PID, and fuzzy neural network PID controller are performed, respectively. The simulation results are shown in Figure 14.

According to the simulation results in Figure 14, the system performance indexes of the three different controllers are summarized in Table 4.

It can be seen from Table 4 that the conventional PID has the largest overshoot, the longest adjustment time, and large fluctuations. Compared with conventional PID, the fuzzy PID has a smaller overshoot and a shorter adjustment time, but the rise time is longer and the fluctuation is smaller. Compared with the previous two methods, the fuzzy neural network PID controller has a faster response, minimum overshoot, the shortest adjustment time, and almost no fluctuation. When disturbed, the overshoot of the conventional PID controller is within 23%, and the system can reach a stable state within 2.88 s. The conventional PID control has a long adjustment time, poor robustness, and weak anti-interference ability. The overshoot of the fuzzy PID controller is within 13% and the fluctuation is not large, but the stability time is as long as 4.25 s and the anti-interference ability is poor. The overshoot of the fuzzy neural network PID controller is within 0.03% and the system can reach a stable state within 1.18 s, which indicates that fuzzy neural network PID controller has a quick response, short adjustment time, small fluctuations, and the strongest anti-interference ability.

## 5. Experimental Verification

Table 5 lists the parameters of the control system based on the system selection, component characteristics, and working environment of the floating body.

**Figure 15:**
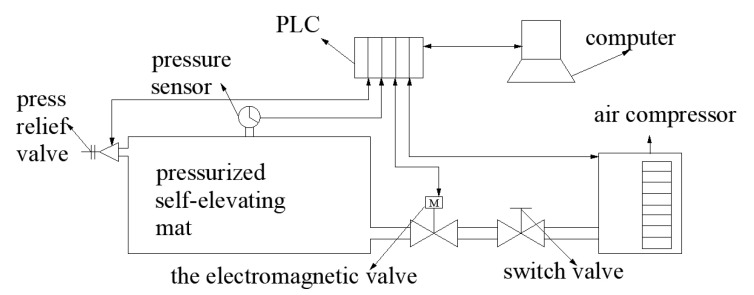
Schematic diagram of pneumatic experiment.

On the basis of the controller design for the self-elevating mat, a self-elevating mat experimental prototype was manufactured. The experimental platform was built to verify the effect of the fuzzy neural network PID in the actual pressure control of a self-elevating mat. Considering the size of the self-elevating mat box and that the focus of this paper was the pneumatic control system, a similar model was adopted for verification. The experimental principle is shown in Figure 15.

**Figure 16:**
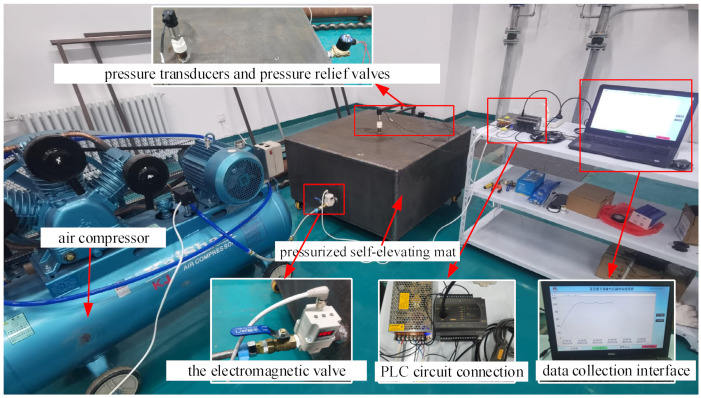
Experimental platform.

The experimental platform mainly included an air compressor, a PLC, a computer, a self-elevating mat, valves, and pipeline components. During the experiment, the required air volume was provided by the air compressor, which was transported into the self-elevating mat through the pipeline valve. The PLC collected the pneumatic values measured by pressure sensors and calculated the opening of the solenoid valve through different controllers for output, so as to realize constant internal pressure in the self-elevating mat. In this experiment, a leakage was set at the pressure relief valve on the self-elevating mat box, and, by using different control logics, we explored the internal pressure conditions during the charging and holding stages of the self-elevating mat when the charging reached 100 kPa. The quality of each control logic was analyzed by collecting data analysis. The experimental platform is shown in Figure 16.

Experimental steps: (1) Firstly, we adjusted the controller logic to the PID controller, opened the pressure relief valve to introduce interference, and started the control system; (2) we started pressurizing and maintaining pressure according to the set pressure, and observed the internal pressure changes on the HMI interface and recorded real-time pressure data; and (3) thirdly, the control logic was adjusted to the fuzzy PID and fuzzy neural PID controller, respectively, and operations (1) and (2) were repeated to record the internal pressure change data under different controllers. (4) Finally, we analyzed and compared the results.

**Figure 17:**
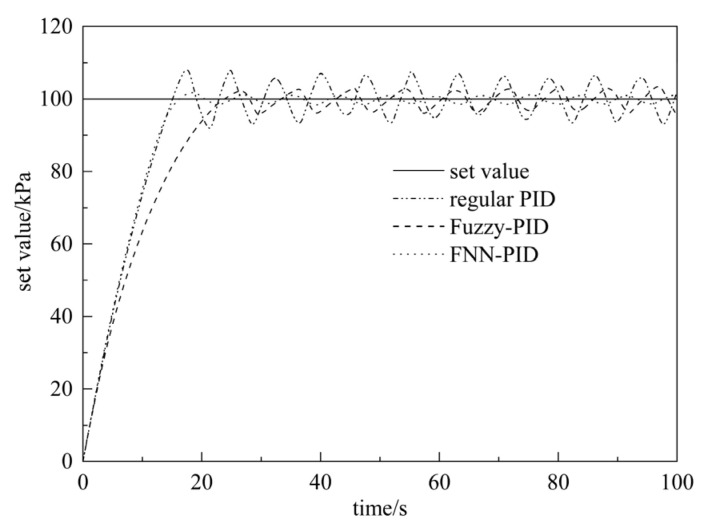
Experimental curve of pressure boost and pressure retention.

According to the pressure experiment procedure, the pressure relief valve was opened to introduce interference, the pressure of the box was pressurized to 100 kPa, and then the pressure was held for a period of time. The pressure data of the self-elevating mat under different controllers were recorded. The experimental curves are shown in Figure 17.

According to the experimental results, it can be seen that the pressure system’s overshoot under the conventional PID controller is the largest, and the fluctuation is relatively severe. The overshoot of the fuzzy PID controller is smaller than that of the conventional PID, but its response speed is too slow. The optimized fuzzy neural network PID controller has excellent performance in reducing the overshoot, speeding up the response, reducing the adjustment time, and enhancing system stability. Due to the structure of the experiment, and certain interference and instability factors present during the experiment process, such as the instability of the output air source pressure of the plunger-type air compressor, the pressure value fluctuated in the pressure holding stage, resulting in a certain deviation between the experimental results and the simulation results.

## 6. Discussion

In this paper, aimed at addressing the problem of insufficient pressure control accuracy of self-elevating mats, the scheme of a self-elevating mat pressure control system is designed, the overall structure of the wind power installation platform is determined, the selection of related equipment in the pressure system is completed, the transfer function of the self-elevating mat pressure control system is established, a fuzzy neural network is used to optimize the fuzzy controller, and the operation monitoring system is designed for the control of the self-elevating mat. Finally, the superiority of the fuzzy neural network PID controller is verified by experiments. Research on the self-elevating mat system’s structure scheme, the pressure model, the control strategy, simulation analysis, hardware and software implementation, etc., provides a theoretical basis and technical guidance for the practical application of the project, but certain aspects still need to be further developed and improved: the air consumption of the self-elevating mat punching pile system has been simplified in the design, the self-elevating mat leakage model and pressure relief valve model are linearized, and the accuracy can be improved. If the design of the self-elevating mat’s pressure control system allows, the experimental verification of the self-elevating mat’s pressure operation monitoring system can be completed on site.

## 7. Conclusions

The pressurized self-elevating mat in this paper is a new type of equipment, and the pneumatic control system for the self-elevating mat is a kind of controlled object that is nonlinear and has a large delay, which is difficult to control. In this paper, the conventional PID controller and fuzzy PID controller are designed, and the PID strategy and fuzzy PID strategy are simulated using Simulink. Comparing the results, it can be seen that the adjustment time and overshoot of the fuzzy PID controller are smaller, but the anti-interference ability is weaker. Therefore, the learning characteristics of the BP neural network are used to optimize the fuzzy PID, and the fuzzy neural network is designed to establish and calculate the membership rule of PID. The simulation results show that the controller optimized using the BP neural network has the best performance with regard to overshoot control, stability time, and anti-interference, which effectively improves the control precision and robustness of the pneumatic control system for the self-elevating mat. Finally, the effectiveness of the simulation results is verified via experiments.

## Figures and Tables

**Table 1:** Δ*K_p_* fuzzy inference table.

*e*	*ec*						
	NB	NM	NS	ZO	PS	PM	PB
NB	PB	PB	PM	PM	PS	ZO	ZO
NM	PB	PB	PM	PS	PS	ZO	NS
NS	PM	PM	PM	PS	ZO	NS	NS
ZO	PM	PM	PS	ZO	NS	NM	NM
PS	PS	PS	ZO	NS	NS	NM	NM
PM	PS	ZO	NS	NM	NM	NM	NB
PB	ZO	ZO	NM	NM	NM	NB	NB

**Table 2:** Δ*K*_i_ fuzzy inference table.

*e*	*ec*						
	NB	NM	NS	ZO	PS	PM	PB
NB	NB	NB	NM	NM	NS	ZO	ZO
NM	NB	NB	NM	NS	NS	ZO	ZO
NS	NB	NM	NS	NS	ZO	PS	PS
ZO	NM	NM	NS	ZO	PS	PM	PM
PS	NM	NS	ZO	PS	PS	PM	PB
PM	ZO	ZO	PS	PS	PM	PB	PB
PB	ZO	ZO	PS	PM	PM	PB	PB

**Table 3:** Δ*K*_d_ fuzzy inference table.

*e*	*ec*						
	NB	NM	NS	ZO	PS	PM	PB
NB	PS	NS	NB	NB	NB	NM	NM
NM	PS	NS	NB	NM	NM	NS	ZO
NS	ZO	NS	NM	NM	NS	NS	ZO
ZO	ZO	NS	NS	NS	NS	NS	ZO
PS	ZO	ZO	ZO	ZO	ZO	ZO	ZO
PM	PB	NS	PS	PS	PS	PS	PB
PB	PB	PM	PM	PM	PS	PS	PB

**Table 4:** System performance specifications.

Controller	Rise Time/s	Adjustment Ime/s	The Peak	Overshoot	Anti-Interference Adjustment Time/s	Disturbance Overshoot
PID	0.875	5.085	13.96	39.6%	2.88	23%
Fuzzy-PID	1.271	4.865	11.07	10.7%	4.25	13%
FNN-PID	1.050	1.514	10.09	0.9%	1.19	0.03%

**Table 5:** Control system parameters.

Parameter Name	Parameter Symbol	Parameter Value	Parameter Unit
Parameter Unit pressure loss coefficient	*ξ*	1.15	—
Pipe density	*ρ*_0_	8.18	kg·m^−3^
Gas flow rate in the pipeline	*μ*	10	m·s^−1^
Total length of straight pipe	*L*_a_	280	m
Lost length of pipeline	*L*_b_	50	m
Total area of intake valves	*A*_1_	0.5	m^2^
Total area of pressure relief valve	*A*_2_	0.2	m^2^
Solenoid valve shape factor	*C*_L_	0.8	—
Adiabatic coefficient	*K*	1.4	—
Operating temperature	*T*	295	K
Volume of floating body	*V*	20,000	m^3^
Gas constant	*R*	8.314	J·(kg·K)^−1^
Air source pressure	*P*_0_	709,275	Pa
Proportional valve amplification factor	*K*_a_	0.8	A·V^−1^
Proportional valve gain	*k_v_*	1.6 × 10^−3^	m·A^−1^
Solenoid valve damping ratio	*ξ_v_*	0.6	—
Natural frequency of solenoid valve	*ω_v_*	120	rad·s^−1^
Pressure sensor coefficient	*K_f_*	1	—

## Data Availability

The data presented in this study are available upon request from the corresponding author.
